# Comparison of WTC Dust Size on Macrophage Inflammatory Cytokine Release *In vivo* and *In vitro*

**DOI:** 10.1371/journal.pone.0040016

**Published:** 2012-07-18

**Authors:** Michael D. Weiden, Bushra Naveed, Sophia Kwon, Leopoldo N. Segal, Soo Jung Cho, Jun Tsukiji, Rohan Kulkarni, Ashley L. Comfort, Kusali J. Kasturiarachchi, Colette Prophete, Mitchell D. Cohen, Lung-Chi Chen, William N. Rom, David J. Prezant, Anna Nolan

**Affiliations:** 1 Division of Pulmonary, Critical Care and Sleep Medicine, New York University School of Medicine, New York, New York, United States of America; 2 Department of Environmental Medicine, New York University School of Medicine, Tuxedo Park, New York, United States of America; 3 Bureau of Health Services and Office of Medical Affairs, Fire Department of New York, Brooklyn, New York, United States of America; 4 Pulmonary Medicine Division, Department of Medicine, Montefiore Medical Center and Albert Einstein College of Medicine, Bronx, New York, United States of America; 5 Ruth L. and David S. Gottesman Institute for Stem and Regenerative Medicine Albert Einstein College of Medicine, Bronx, New York, United States of America; Louisiana State University, United States of America

## Abstract

**Background:**

The WTC collapse exposed over 300,000 people to high concentrations of WTC-PM; particulates up to ∼50 mm were recovered from rescue workers’ lungs. Elevated MDC and GM-CSF independently predicted subsequent lung injury in WTC-PM-exposed workers. Our hypotheses are that components of WTC dust strongly induce GM-CSF and MDC in AM; and that these two risk factors are in separate inflammatory pathways.

**Methodology/Principal Findings:**

Normal adherent AM from 15 subjects without WTC-exposure were incubated in media alone, LPS 40 ng/mL, or suspensions of WTC-PM_10–53_ or WTC-PM_2.5_ at concentrations of 10, 50 or 100 µg/mL for 24 hours; supernatants assayed for 39 chemokines/cytokines. In addition, sera from WTC-exposed subjects who developed lung injury were assayed for the same cytokines. In the *in vitro* studies, cytokines formed two clusters with GM-CSF and MDC as a result of PM_10–53_ and PM_2.5_. GM-CSF clustered with IL-6 and IL-12(p70) at baseline, after exposure to WTC-PM_10–53_ and in sera of WTC dust-exposed subjects (n = 70) with WTC lung injury. Similarly, MDC clustered with GRO and MCP-1. WTC-PM_10–53_ consistently induced more cytokine release than WTC-PM_2.5_ at 100 µg/mL. Individual baseline expression correlated with WTC-PM-induced GM-CSF and MDC.

**Conclusions:**

WTC-PM_10–53_ induced a stronger inflammatory response by human AM than WTC-PM_2.5_. This large particle exposure may have contributed to the high incidence of lung injury in those exposed to particles at the WTC site. GM-CSF and MDC consistently cluster separately, suggesting a role for differential cytokine release in WTC-PM injury. Subject-specific response to WTC-PM may underlie individual susceptibility to lung injury after irritant dust exposure.

## Introduction

The destruction of the World Trade Center (WTC) led to the release of an estimated 10 million tons of dust, exposing over 300,000 rescue workers and New York City (NYC) residents to WTC particulate matter (WTC-PM).[1]–[4] The concentrations of airborne and respirable WTC-PM ranged from 1–100 mg/m^3^.[5]–[7]. Many rescue and recovery workers continued to be exposed to dust for at least three months during the clean-up and recovery phase. [8] The toxicology and physical properties of WTC-PM are well described. [3], [6] Bulk WTC-PM is composed of pulverized concrete, plastics, other building materials and combustion by-products (hydrocarbons, etc. [6] WTC-PM was found to be highly alkaline: pH 9–11. [6], [9] The size of WTC-PM ranged from PM_2.5_; ≤2.5 µm to PM_53_; ≥1–53 µm.

Epidemiologic evidence links PM exposure to hospitalization and mortality from cardiovascular and pulmonary diseases. [10]–[12] Acute airway inflammation has been described after exposure to ambient PM and WTC-PM. [7], [13]–[15] Exposure to WTC-PM has been implicated in the development of lung injury, reactive airways’ dysfunction, obstructive airway physiology and overall decline in FEV_1_. [2], [16].

Alveolar macrophages are a primary cell type that interacts with inhaled particulates, and are intimately involved in the elaboration of the lung’s inflammatory response. Resident macrophages interact with the acute phase neutrophils that migrate into the alveolar space during inflammation. The initial activation of pattern recognition receptors (PRR), such as Toll-Like Receptor (TLR)-4, results in production of chemokines and cytokines which amplify the existing inflammatory response and recruit additional inflammatory cells including neutrophils. [17] WTC-PM exposure in a murine model caused airway hyperresponsiveness and neutrophils infiltration as measured by BAL. [18] Previous studies have shown that human alveolar macrophages (AM) and epithelial cells exposed to WTC-PM at doses of 5 and 50 µg, led to an increased production of interleukin (IL)-8 and IL-6. However, a 10-fold increased dose of WTC-PM led to a decline in production of these same cytokines. [19] Human fibroblasts exposed to WTC-PM had decreased cell proliferation and increased apoptosis. [20].

In a recent study of serum biomarkers in FDNY workers, an elevated Granulocyte Macrophage-Colony Stimulating Factor (GM-CSF) and Macrophage Derived Chemokine (MDC) within 5 months of 9/11 increased the odds of developing abnormal lung function in the next 6.5 years. [21] Roles for GM-CSF and MDC in airway injury are biologically plausible since GM-CSF is elaborated by macrophages causing Th-2 polarization during antigen presentation in asthma. [22] In addition, human bronchial epithelial cells produce GM-CSF in response to PM_2.5._ [23]–[25] MDC (CCL22) is elevated in models of tobacco-induced lung injury and may be responsible for recruiting inflammatory cells to the lung. [26].

In WTC-exposed NYC firefighters, bronchoalveolar lavage obtained 1 month post-exposure [13] and induced sputum obtained 10 months post-exposure [27] showed increased small and large dust particles (PM_1–50_), neutrophils and eosinophils. Acknowledging that large particles PM_10–53_ did enter the small airways, we examined the effects of WTC-PM_10–53_ in comparison to WTC-PM_2.5_. This study indicates that WTC-PM_10–53_ more strongly stimulates alveolar macrophages exposed *in vitro* to produce GM-CSF and MDC. Clustering of analytes *in vivo* and *in vitro* suggests that GM-CSF and MDC are in separate inflammatory pathways that can produce airway injury after WTC-PM exposure.

## Methods

### WTC Particulates

#### Collection

The WTC-PM were collected in bulk from site #13 (Liberty and Church Street, 0.1 miles southeast of Ground Zero), the particles were aerosolized, sieved using a 53-µm diameter mesh screen and size separated by using a 10-µm cut Wedding inlet (Anderson Instrument Co, NY) to isolate PM_<10_ and PM≥_10_ fractions. [6] The PM_<10_ fractions were passed through a 2.5-µm cutting inlet to isolate WTC-PM_2.5_ fractions on Teflon filters. The WTC-PM_2.5_ and WTC-PM_10–53_ dusts were stored in a dark environment at room temperature as recommended by Drs. Lung-Chi Chen and Mitchell Cohen who kindly provided these samples. No specific permits were required for the described field studies and the collection site was not privately-owned.

#### Extraction

WTC-PM_10–53_ samples were weighed and suspended in PBS; WTC-PM_2.5_ was extracted from Teflon filters in sterile PBS (1 mL) under sonication. [6] Filters were weighed before and after the particles were extracted. Lyophilized samples were re-suspended in sterile PBS. The final concentration of particles in solution was then adjusted to 0.5 µg/mL and aliquots of samples were stored at −40°C until utilized.

#### WTC-PM stock solution endotoxin content and pH assessment

WTC-PM_2.5_ was prepared in a 0.5 mg/mL stock in sterile PBS and had a pH of 9.8 and an endotoxin level of 0.65 EU/mL; WTC-PM_10–53_ (1 mg/mL stock) in sterile PBS had a pH of 10.2 and an Endotoxin level of 0.63 EU/mL, which is consistent with earlier levels of measurement. [6].

### Bronchoscopy

The bronchoalveolar lavage (BAL) protocol was approved by the human subjects review committees of New York University Medical Center Institutional Review Board and by Bellevue Hospital Center Research Review Committee (H09-0769, 07-601 and 3165). Human subjects without WTC-exposure or pulmonary symptoms and with normal chest radiographs signed informed consent at the time of enrollment allowing analysis of their information and samples for research. Volunteers underwent bronchoscopy with BAL with 120 mL saline. Briefly, lavage was filtered through two layers of sterile cotton gauze to remove mucus and re-suspended with supplemented media. [28], [29].

### Cell Culture

BAL (n = 15 subjects) cells were plated at 1x10^6^ cells/mL overnight in RPMI media 1640 (Gibco, Grand Island, NY) with 10% fetal calf serum (HyClone, Logan, UT), 2% Penicillin-Streptomycin (Gibco, USA) in 12-well plates. After 24 hours, non-adherent cells and culture media were discarded. Fresh culture media was added, and adherent macrophages were exposed to 10, 50 or 100 µg/mL suspensions of WTC-PM_2.5_ and WTC-PM_10–53_. Media alone was the negative control and 40 ng of Lipopolysaccharide (LPS)/mL (E. Coli 0.55:B4 and B5, Sigma-Aldrich, St. Louis, MO) was the positive control. After 24 hours, supernatants were collected and assayed using Human Cytokine Panel I (Millipore, Billerica, MA) according to manufacturer’s instructions in a Luminex 200IS (Luminex Corp, Austin, TX). Data were analyzed with MasterPlex TM QT software (Ver. 1·2; MiraiBio, Inc. Alameda, CA). The dynamic range of the assay was defined by the manufacturer. Cell viability was assessed using Trypan blue staining (Invitrogen, Carlsbad, CA) as previously described. [30].

### *In Vivo* WTC-Exposed Subjects

FDNY firefighters (N = 1720) with respiratory symptoms were entered into the Medical Monitoring and Treatment Program, and were further referred to subspecialty pulmonary evaluation. [31] Never smoker males with reliable NHANES FEV_1_ measurement and a pre-9/11 FEV_1_%Predicted ≥75% (N = 801/1720) were part of a nested case control study. Cases were defined as those with a FEV_1_% in the bottom octile of the cohort and with biomarker data available (N = 70/100). Cases had airflow obstruction, and were below the LLN at the time of subspecialty evaluation. Controls were subjects randomly selected (N = 138) from the parent cohort N = 801. This reference group represents analyte expression in the parent cohort. The subject’s demographics, and the measurement of serum inflammatory cytokines by Luminex 200IS (Luminex Corporation, Austin, TX) were previously described. [21] Of the 194 studied with cytokine data, 70 had abnormal lung function upon presentation for treatment of lung injury over the subsequent 6.5 years. [21], [31], [32].

### Statistical Analysis

Data base management and statistics were performed with SPSS 19 (IBM, Armonk, NY) and GraphPad Prism 5.0 (GraphPad, San Diego, CA). Luminex data was analyzed using MasterPlex QT (Ver.1.2; MiraiBio). Normally distributed data were expressed as means and standard deviation. Levels of analyte expressed after exposure to WTC-PM_10–53_ and WTC-PM_2.5_ at each dose were compared by Wilcoxon Signed Rank test, and significance assessed by p<0.05. To test for confounding, baseline analyte levels were compared against race, gender, and smoking status by Kruskal-Wallis one-way ANOVA. Pearson’s correlation was used to compare age and analyte levels at baseline. Spearman’s Rank Correlation assessed subject-specific dose response.

Baseline expression of analytes was examined by hierarchical clustering for both our in vitro analytes and serum cytokine data from our 70 firefighters with abnormal lung function (Cluster 3.0; Ver.1.47, Michael Eisen: Stanford University, Michiel de Hoon: University of Tokyo). [21] Variables were adjusted by log transformation and centered on the median. Similarity was assessed by Spearman Rank Correlation and average linkage, and characterized by a dendrogram using Java Treeview; Ver.1.1. [33], [34] Spearman rank correlation was utilized to allow for analysis of nonparametric data, and to capture nonlinear monotonic relationships between the analytes. [35].

## Results

### Alveolar Macrophage Cytokines Released *in vitro*

Human subjects (N = 15) without WTC-exposure or pulmonary symptoms and with normal chest radiographs had research bronchoscopy and BAL to retrieve resident immune cells from the lung. The volunteers were predominantly male (73%, N = 11/15), had mean (SD) age of 52(10) and BMI of 29.0(6.0), Table 1. BAL contained 91% AM, 7% lymphocytes, 2% neutrophils and 0% eosinophils. After adherence, over 99% of the cells were viable and had macrophage morphology. After 24 hours *in vitro* culture, supernatants were recovered and assayed for 39 cytokines and chemokines using a multiplex assay.

**Table 1:** BAL Differential and Demographics of Study Population.

Subject	Differential*				Gender	Race	Ever Smoker	Age	BMI
	M	L	N	E					
1	98	0	2	0	M	AA	Y	48	34.4
2	95	4	1	0	F	AA	N	52	28.1
3	96	1	3	0	M	AA	Y	60	40.1
4	85	14	1	1	M	C	Y	54	27.4
5	86	12	2	0	M	C	N	29	27.7
6	83	14	2	0	M	C	N	57	32.4
7	96	2	2	0	M	C	Y	67	27.0
8	86	13	1	0	M	C	N	37	23.7
9	91	6	3	0	M	AA	N	42	34.7
10	92	7	1	0	F	AA	Y	53	27.4
11	95	3	2	0	M	C	Y	61	23.3
12	90	8	1	1	M	C	Y	52	23.7
13	86	12	2	0	F	C	Y	57	27.2
14	92	6	2	0	M	C	Y	53	37.2
15	95	4	1	0	F	C	Y	63	21.2
Cumulative	91(5)	7(5)	2(1)	0(0)	73.3(11)**	66.7(10)**	66.7(10)**	52 (10) #	29.0(6)#

**Abbreviations:** M-Macrophages; L-Lymphocytes; N-Neutrophils; E-Eosinophils; M-Male; F-Female; AA-African American; C-Caucasian; Y-Yes, Ever-Smoker; N-No, Never-Smoker.

* Differential rounded to whole number, Cumulative expressed as Mean (SD).

** Cumulative expressed as %(N): Male/Caucasian/Ever-Smoker.

# Cumulative expressed as Mean (SD).

AM cultured in media alone expressed 22/39 analytes greater than the limit of detection (LOD) while 17/39 analytes had median baseline expression below LOD (Table S1A and S1B). Figure l shows the dose response for GM-CSF, IL-6, IL-10, and tumor necrosis factor (TNF-α), demonstrating the maximal response at 10, 50 and 100 µg/mL and that PM_10–53_ was significantly greater than PM_2.5_ for each dose and cytokine. The 100 µg/mL dose of WTC dust was used for all additional analyses (see Table S1A and S1B for responses for all doses). AM exposed to media alone released low levels of cytokines, which were increased significantly with LPS (positive control), Table 2. Increased cytokine release was noted for GM-CSF, TNF-α, IL-6, IL-7, IL-10, IL-12(p70), and interferon (IFN)-γ when comparing PM_10–53_ to PM_2.5_, Table 2. Among these cytokines, release was lower for PM_10–53_ than the positive control LPS with the exception of GM-CSF (372 vs 268 pg/mL by LPS; p<0.05), Table 2. Other cytokines that were increased by PM_10–53_ compared to PM_2.5_ were: IL-1α, MDC, and MCP-3. Importantly, IL-8 was strikingly increased by all levels of dust and LPS compared to media alone.

**Figure 1:**
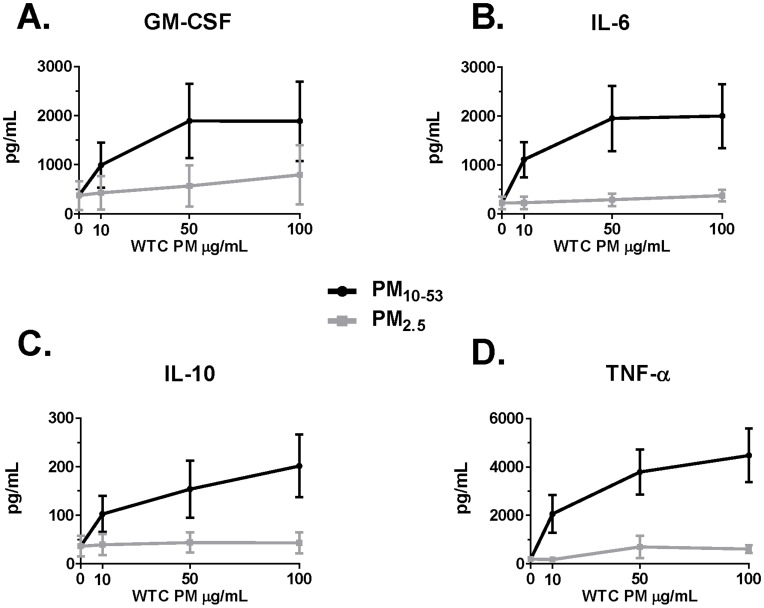
Cytokine expression by Alveolar Macrophages 24 hours after stimulation with increasing doses of WTC-PM_2.5_ and WTC-PM_10–53_ in µg/ml. All cytokines with a ≥2-fold induction of PM_10–53_/PM_2.5_ at each dose are shown. All graphs show mean ± SEM of cytokine, p<0.01 for all comparisons between PM_10–53_ and PM_2.5_ by Wilcoxon Matched Pairs Signed Rank Test. Panel A) GM-CSF B) IL-6 C) IL-10 D) TNF-α. N = 14 for WTC-PM_2.5_ and WTC-PM_10–53_ at 10 µg/mL; N = 15 for all other exposures.

**Table 2:** *In Vitro* Expression of Chemokines and Cytokines by Alveolar Macrophages*.

Analyte	Media Alone		WTC PM_2.5_ 100 µg/mL	WTC PM_10–53_ 100 µg/mL	LPS 40 ng/mL
	Median (IQR)	CV(%)			
**Eotaxin**	24.6 (17–41)	59	38.5(26–46)	37.8(23–45)#	41.2(28–51)
**GM-CSF**	16.4(7–66)	302	69.7(45–235)**	371.5(149–1082)^##^	267.5(163–1224)
**IL-6**	32.0(8–62)	218	239.2(131–489)**	1409.6(404–2357)	6148.2(1978–>10000)
**TNF-α**	31.6(13–142)	203	340.8(233–688)**	1784.0(1209–9314)	7852.7(1803–>10000)
**Fractalkine**	33.5(30–69)	89	88.8(54–112)	147.6(63–192)	253.7(150–292)
**IFNα-2**	16.2(7–29)	122	35.0(20–42)	42.7(18–49)	46.4(35–112)
**IL-12(p70)**	3.4(<3.2–8)	90	6.8(<3.2–9)**	9.0(5–10)	11.1(7–20)
**IL-10**	11.1(<3.2–20)	227	27.7(8–36)**	100.6(29–238)	484.0(272–1186)
**IFN-γ**	12.2(<3.2–16)	311	26.2(11–33)**	36.0(17–62)	53.5(37–86)
**IL-7**	20.7(<3.2–37)	104	32.5(12–63)**	66.7(41–107)	97.0(66–131)
**VEGF**	51.4(23–105)	90	155.2(55–205)	143.6(59–206)	208.7(80–239)
**Flt-3-Ligand**	10.4(5–14)	99	17.0(9–38)	12.9(7–35)#	21.4(6–46)
**IL-1α**	4.3(<3.2–29)	137	14.3(6–39)**	40.2(8–72)#	49.8(7–75)
**MDC**	1803.0(483–3348)	116	3236.7(974–5234)**	5008.6(62->10000)#	7088.5(1193–>10000)
**IL-1ra**	177.5(43–751)	212	404.3(65–1183)	360.5(52–1235)#	412.7(55–1169)
**MCP-3**	221.9(95–309)	134	86.5(23–282)**	196.4(21–422)	635.0(115–3224)
**IP-10**	53.8(20–1215)	210	28.2(14–311)	136.2(33–290)	2587.1(39->10000)
**IL-8**	3081.1(1643–9115)	80	>10000(2158–>10000)	>10000(2535–>10000)#	>10000(2082–>10000)
**GRO**	187.3(97–668)	248	500.5(309–2297)	600.2(75->10000)#	8212.1(385->10000)
**MIP-1β**	39.1(14–131)	199	647.1(334–1619)	273.9(15–3528)	8662.0(1199–>10000)
**MIP-1α**	96.6(44–824)	218	2920.1(769->10000)	3028.2(43->10000)#	>10000(206->10000)
**MCP-1**	2230.9(1398–3138)	62	2646.8(2049–4777)	2726.2(2093–4160)	3256.1(2129–7128)

* Analyte (N = 22) levels in pg/mL, expressed as Median (IQR). Analytes above Solid Line are in the cluster containing GM-CSF. Analytes below Solid Line are in the cluster containing MDC.

Following calculated by Wilcoxon Paired Signed Rank Test:

** PM_2.5_ significantly Less than PM_10–53_, p<0.05;

# PM_10–53_ Not Significantly Different from LPS 40 ng/mL and ^##^Greater than LPS 40 ng/mL, p<0.05.

**Figure 2:**
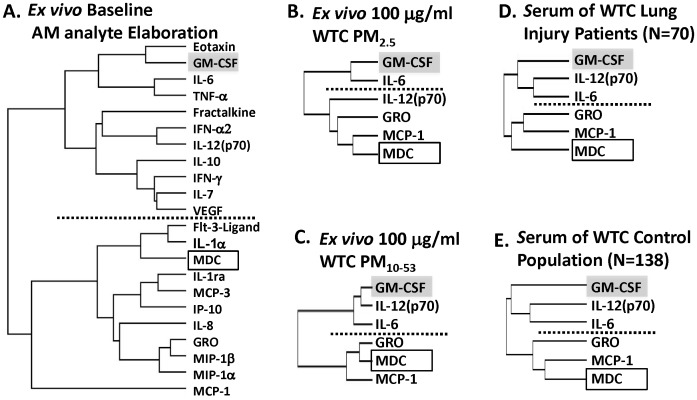
Hierarchical Clustering of Chemokine and Cytokine Expression in in vitro and in vivo exposures to WTC-PM. Clustering was performed by log-transformed data and Spearman Correlation, with average linkage. GM-CSF is highlighted in grey, and MDC is highlighted in the clear box. Shown is the clustering of chemokines and cytokines that were consistently segregated in the *in vitro* and *in vivo* studies. GM-CSF and MDC are in separate clusters. **A**) The 22 analytes with median baseline expression levels above the LOD were clustered. Two separate clusters were identified, each containing 11 analytes. *In vitro*, baseline **B**) WTC-PM_2.5_ 100 µg/mL *in vitro*. **C**) WTC-PM_10–53_ 100 µg/mL in vitro. **D**) Serum from WTC-exposed patients with WTC-Lung Injury. E) Serum from the WTC-Exposed control population with normal lung function.

We performed hierarchical clustering of cytokine and chemokine elaboration to identify analytes with related patterns of expression, Figure 2. At baseline AM elaborated analytes that segregated into two clusters, each contains 11 analytes, Figure 2A. GM-CSF clustered with IL-12 and IL-6 while MDC clustered with MCP-1 and GRO chemokines, Figure 2A. Clustering of analyte expression from AM after WTC-PM_2.5_ and WTC-PM_10–53_ consistently demonstrated two clusters of expression with GM-CSF segregating into a different cluster than MDC, Figure 2B and C.

### Serum Cytokines Released *in vivo*

To test if *in vitro* expression patterns reflected cytokine and chemokine production *in vivo*, we repeated clustering of analyte expression in serum drawn from WTC exposed rescue workers (n = 70) within 6 months of 9/11/2001. [21] As with dust stimulated AM, serum GM-CSF clustered with IL-12 and IL-6 while MDC clustered with MCP-1 and GRO chemokines, Figure 2D. To test if segregation of MDC and GM-CSF expression in serum is due to selection bias produced by the case definition we repeated the hierarchical clustering using serum of control subjects. There was no difference analyte clustering in cases and controls, Figure 2E.

### WTC-PM_10–53_ Stimulates Inflammatory Chemokine and Cytokine Release More Strongly than WTC-PM_2.5_

WTC-PM_10–53_ induced greater analyte expression than WTC-PM_2.5,_ for most analytes, Table 2. WTC-PM_10-53_ induced at least two fold more GM-CSF, IL-6, TNF-α, and IL-10 than WTC-PM_2.5_ at all doses tested (p<0.01 by Wilcoxon Matched Pairs Signed Rank Test, Figure 1 and Table S1A. WTC-PM_10–53_ at 100 µg/ml induced Eotaxin, Fractalkine, IL-1α, IL-1ra, MDC, MIP 1-α, and IL-8 to the same extent as LPS. WTC-PM_10–53_ induced 13 analytes less strongly than did LPS. At baseline and at all dust doses EGF, IL-4, IL-5, IL-9, IL-13, IL-15, IL-17, TGF-α, sCD40L and TNF-β were below the limits of detection even though over 80% of cells were viable 24 hours after exposure to WTC dusts, Table S1B. IL-3, IL-5, IL-9, IL-15 and EGF did not respond to any PM stimulation but also did not respond to 40 ng/ml of LPS, Table S1B.

**Subject-Specific response to WTC-PM_10–53_ and PM_2.5_ correlated with baseline release.** Individuals with elevated serum GM-CSF and MDC had higher odds of developing abnormal lung function after exposure to dust at the WTC site. AM exposed to media alone demonstrated inter-subject variation in baseline chemokine and cytokine elaboration. The inter-person variability of expression at baseline was assessed by the coefficient of variation (CV%), which ranged from 59% to 311%, Table 2. To test if baseline GM-CSF and MDC release correlated with induction by WTC dust, we used a Spearman’s Rank Correlation to test if there is a subject-specific response to WTC dust exposure. There was high concordance between baseline and WTC-PM_2.5_ induced GM-CSF and MDC at all dust concentrations (range 0.799-0.960, p<0.01 for all doses), Table 3. Concordance between baseline release and WTC-PM_10–53_ stimulated release was less robust, reaching significance at 100 µg/ml for both analytes (range 0.536–0.614, p<0.05).

**Table 3:** Spearman’s Rank Order Correlation Coefficients Relative to Unstimulated Baseline.

PM Dose	GM-CSF		MDC	
	PM_10–53_	PM_2.5_	PM_10–53_	PM_2.5_
**10**	.429	.938**	.719**	.960**
**50**	.450	.873**	.782**	.799**
**100**	.614*	.821**	.536*	.807**

Particulate Matter (PM) in µg/mL;

* p<0.05.

** p<0.01.

## Discussion

FDNY workers who had elevated serum GM-CSF months after 9/11/2001 had a 2.5 fold increase in the odds ratio of developing abnormal lung function within the next 6.5 years. Similarly, elevated serum MDC independently increased the odds of developing abnormal FEV_1_ by 2.95-fold. Bronchoalveolar lavage done several weeks after 9/11 demonstrated 8–50 µm particles within alveolar macrophages. [27] Sputum induced 9 months later contained dust ranging from 1 to 50 µm in diameter. [27] This massive dust exposure overcame the normal protective mechanisms that prevent large particles from entering the lower airway and resulted in a significant inflammatory response.

Since particles up to 50 microns in size were recovered from the lungs of WTC-exposed firefighters 1 to 10 months after 9/11/2001, we examined the inflammatory response of AM to both WTC-PM_10–53_ and WTC-PM_2.5_. WTC-PM_10–53_ 100 µg/mL induced significantly more inflammatory mediators than WTC-PM_2.5_ in 12/22 analytes with measurable baseline expression. WTC PM_10–53_ induced greater release of GM-CSF, IL-6, TNF-α, and IL-10 than WTC-PM_2.5_ for all doses tested. Surprisingly, WTC-PM_10–53_ 100 µg/mL induced more GM-CSF than LPS 40 ng/mL. WTC-PM_10–53_ contains particles too large for effective phagocytosis. The strong inflammatory response to stimulation with large particles may be due to frustrated phagocytosis. [36] Since 100 µg of the dust preparations had less than 0.02 ng of LPS, it is unlikely that bacterial contamination drove the inflammatory response. The intense inflammatory response produced by WTC-PM_53_ may explain why 19% of the 2,152 rescue workers caught in the dust cloud at the time of the collapse needed subspecialty pulmonary evaluation and treatment over the next 6.5 years. [31].

Our findings on spontaneous release and PM_2.5_ induction of GM-CSF are similar to other large series of human AMs obtained from lobectomy samples, cultured *ex vivo* and stimulated with PM_10_. [37] Other studies on WTC-PM stimulation of AM used a small number of AM and measured IL-6 and TNF-α production. [19], [20] These cytokines activate other inflammatory cells by stimulating lung epithelium. [38] In contrast, our work has used bronchoscopy to obtain cells and has a sufficient number of AM samples to portray subject-specific variability of cytokine/chemokine expression.

Hierarchical clustering demonstrated that MDC and GM-CSF segregated into separate clusters, in both AM preparations *in vitro* and in serum from WTC dust-exposed firefighters who progressed to abnormal lung function. This reflects our previous findings that MDC and GM-CSF are independent predictors of lung injury in the FDNY WTC-exposed firefighters. [21] Clustering of previously quantified GM-CSF showed that it co-segregated with IL-6 and IL-12(p70) indicating coordinated expression of macrophage derived cytokines *in vivo* in patients after WTC dust exposure and *in vitro* in AM exposed to WTC-PM_10–53_. Similarly, MDC clustered with GROα and MCP-1 after WTC-PM exposure *in vivo* and *in vitro* indicating coordinated regulation of these macrophage derived chemokines. Further research is needed to understand the mechanism of coordinated regulation of these cytokines and chemokines.

Our observations are consistent with other reports that GM-CSF is up-regulated by fine, ultrafine, and other intermediate ambient particulates. [37], [39] GM-CSF mediates the effects of airway inflammation in a murine model. [40] GM-CSF activity in airway injury is biologically plausible since human bronchial epithelial cells produce GM-CSF *in vitro* in response to PM_2.5_. [23]–[25] This suggests a role for GM-CSF in inflammation produced by lung epithelium in diseases that cause airflow obstruction. The profound effects of large particulates on GM-CSF induction by AM in addition to the observation that PM remains in the lower airway long after exposure provides a framework for understanding why persistent inflammation as evidenced by high serum GM-CSF is a risk factor for subsequent abnormal FEV_1_ in the 6.5 years following 9/11/2001. The amount of MDC and GM-CSF produced by PM_2.5_ was correlated with the baseline factor expression. Subjects with high baseline GM-CSF had greater dust-induced cytokine production. This finding was not dependent upon gender, age, race or smoking status. This may indicate individual predisposition to lung injury is reflected by subject-specific spontaneous cytokine release.

This study has several limitations. The cultured AM were obtained from subjects who did not have WTC exposure. WTC-exposed FDNY personnel with low FEV_1_ are at increased risk for complications from a research bronchoscopy. We are therefore unable to correlate subjects’ AM baseline release of GM-CSF and MDC with their susceptibility to lung injury following WTC exposure. As a result, we cannot test the hypothesis that patients with high spontaneous factor release by AM are more susceptible to inflammatory injury. We also did not investigate the underlying mechanisms of individual variation in cytokine production or why GM-CSF and MDC independently regulated.

Our findings of similar chemokine and cytokine clustering in serum of WTC exposed patients suggests that out *in vitro* conditions accurately represent patterns of analyte expression as observed in vivo. The AM preparations in this study were cultured *in vitro* for 24 hours. This will alter the AM behavior in unpredictable ways. For example, the bronchoscopically obtained cells were washed prior to plating, reducing surfactant protein concentration during the adherence and exposure period. This could alter IL-6 production by macrophages because surfactant protein A is an inhibitor. [41] Thus, factor release in media alone conditions will be different from *in situ* AM.

Our findings of similar chemokine and cytokine clustering in serum of WTC dust-exposed patients suggests that our *in vitro* experiments likely reflect patterns of analyte expression observed *in vivo*. This finding is consistent with AM being a major source of GM-CSF and MDC in the serum of WTC exposed firefighters. Several WTC-lung injury subgroups exist in a narrowly focused group of symptomatic firefighters with high WTC dust-exposure. It is unclear why these individuals have differing lung injury patterns. Our work has tried to explore two possible causes. First, individuals expressing varying innate mediator elaboration could identify and associate with an increased susceptibility to lung injury. Second, the different response to WTC-PM_10–53_ and WTC-PM_2.5_ exposure may indicate a different pattern or mechanism to injury. This is consistent with the hypothesis that dust exposed AM have at least two independent inflammatory pathways producing lung injury, one represented by MDC and the other by GM-CSF. Future studies will explore the mechanisms behind the varying spontaneous production of these cytokines and chemokines. Understanding the role of these biomarkers in PM-induced lung injury is necessary to develop individualized therapeutic strategies for future populations with high PM exposure.

## Supporting Information

Table S1 **Expression of Analytes by Alveolar Macrophages for All Stimuli A.** Baseline Analyte Expression Above Limits of Detection (LOD) **B**. Baseline Analyte Expression Below LOD
(DOCX)

## References

[1] Landrigan PJ (2001). Health consequences of the 11 September 2001 attacks.. Environ Health Perspect.

[2] Aldrich TK, Gustave J, Hall CB, Cohen HW, Webber MP (2010). Lung function in rescue workers at the World Trade Center after 7 years.. N Engl J Med.

[3] Lioy PJ, Weisel CP, Millette JR, Eisenreich S, Vallero D (2002). Characterization of the dust/smoke aerosol that settled east of the World Trade Center (WTC) in lower Manhattan after the collapse of the WTC 11 September 2001.. Environ Health Perspect.

[4] Wang RY (2008). Medical toxicology and public health-update on research and activities at the Centers for Disease Control and Prevention and the Agency for Toxic Substances and Disease Registry.. J Med Toxicol.

[5] Chen LC, Thurston G (2002). World Trade Center cough.. Lancet.

[6] McGee JK, Chen LC, Cohen MD, Chee GR, Prophete CM (2003). Chemical analysis of World Trade Center fine particulate matter for use in toxicologic assessment.. Environ Health Perspect.

[7] Prezant DJ, Weiden M, Banauch GI, McGuinness G, Rom WN (2002). Cough and bronchial responsiveness in firefighters at the World Trade Center site.. N Engl J Med.

[8] Landrigan PJ, Lioy PJ, Thurston G, Berkowitz G, Chen LC (2004). Health and environmental consequences of the world trade center disaster.. Environ Health Perspect.

[9] Lioy PJ, Pellizzari E, Prezant D (2006). The World Trade Center aftermath and its effects on health: understanding and learning through human-exposure science.. Environ Sci Technol.

[10] Samet JM, Dominici F, Curriero FC, Coursac I, Zeger SL (2000). Fine particulate air pollution and mortality in 20 U.S. cities, 1987–1994.. N Engl J Med.

[11] Thurston GD, Ito K, Hayes CG, Bates DV, Lippmann M (1994). Respiratory hospital admissions and summertime haze air pollution in Toronto, Ontario: consideration of the role of acid aerosols.. Environ Res.

[12] Dockery DW, Pope CA, 3rd, Xu X, Spengler JD, Ware JH, et al (1993). An association between air pollution and mortality in six U.S. cities.. N Engl J Med.

[13] Rom WN, Weiden M, Garcia R, Yie TA, Vathesatogkit P (2002). Acute eosinophilic pneumonia in a New York City firefighter exposed to World Trade Center dust.. Am J Respir Crit Care Med.

[14] Ghio AJ, Kim C, Devlin RB (2000). Concentrated ambient air particles induce mild pulmonary inflammation in healthy human volunteers.. Am J Respir Crit Care Med.

[15] Ghio AJ, Devlin RB (2001). Inflammatory lung injury after bronchial instillation of air pollution particles.. Am J Respir Crit Care Med.

[16] Banauch GI, Alleyne D, Sanchez R, Olender K, Cohen HW (2003). Persistent hyperreactivity and reactive airway dysfunction in firefighters at the World Trade Center.. Am J Respir Crit Care Med.

[17] Medzhitov R (2001). Toll-like receptors and innate immunity.. Nat Rev Immunol.

[18] Gavett SH (2003). World Trade Center fine particulate matter–chemistry and toxic respiratory effects: an overview.. Environ Health Perspect.

[19] Payne JP, Kemp SJ, Dewar A, Goldstraw P, Kendall M (2004). Effects of airborne World Trade Center dust on cytokine release by primary human lung cells in vitro.. J Occup Environ Med.

[20] Lambroussis C (2009). Indications of Potential Toxic/Mutagenic Effects of World Trade Center Dust on Human Lung Cell Cultures.. Online Journal of Biological Sciences.

[21] Nolan A, Naveed B, Comfort AL, Ferrier N, Hall CB (2011). Inflammatory Biomarkers Predict Airflow Obstruction after Exposure to World Trade Center Dust. Chest..

[22] Bleck B, Tse DB, Jaspers I, Curotto de Lafaille MA, Reibman J (2006). Diesel exhaust particle-exposed human bronchial epithelial cells induce dendritic cell maturation.. J Immunol.

[23] Liu L, Jarjour NN, Busse WW, Kelly EA (2004). Enhanced generation of helper T type 1 and 2 chemokines in allergen-induced asthma.. Am J Respir Crit Care Med.

[24] Reibman J, Hsu Y, Chen LC, Kumar A, Su WC (2002). Size fractions of ambient particulate matter induce granulocyte macrophage colony-stimulating factor in human bronchial epithelial cells by mitogen-activated protein kinase pathways.. Am J Respir Cell Mol Biol.

[25] Hartl D, Griese M, Nicolai T, Zissel G, Prell C (2005). Pulmonary chemokines and their receptors differentiate children with asthma and chronic cough.. J Allergy Clin Immunol.

[26] Ritter M, Goggel R, Chaudhary N, Wiedenmann A, Jung B (2005). Elevated expression of TARC (CCL17) and MDC (CCL22) in models of cigarette smoke-induced pulmonary inflammation.. Biochem Biophys Res Commun.

[27] Fireman EM, Lerman Y, Ganor E, Greif J, Fireman-Shoresh S (2004). Induced sputum assessment in New York City firefighters exposed to World Trade Center dust.. Environ Health Perspect.

[28] Weiden M, Tanaka N, Qiao Y, Zhao BY, Honda Y (2000). Differentiation of monocytes to macrophages switches the Mycobacterium tuberculosis effect on HIV-1 replication from stimulation to inhibition: modulation of interferon response and CCAAT/enhancer binding protein beta expression.. J Immunol.

[29] Gold JA, Hoshino Y, Hoshino S, Jones MB, Nolan A (2004). Exogenous gamma and alpha/beta interferon rescues human macrophages from cell death induced by Bacillus anthracis.. Infect Immun.

[30] Kobayashi H, Nolan A, Naveed B, Hoshino Y, Segal LN (2011). Neutrophils Activate Alveolar Macrophages by Producing Caspase-6-Mediated Cleavage of IL-1 Receptor-Associated Kinase-M.. Journal of Immunology.

[31] Weiden MD, Ferrier N, Nolan A, Rom WN, Comfort A (2010). Obstructive airways disease with air trapping among firefighters exposed to World Trade Center dust.. Chest.

[32] Naveed B, Weiden MD, Kwon S, Gracely EJ, Comfort AL (2012). Metabolic syndrome biomarkers predict lung function impairment: a nested case-control study.. Am J Respir Crit Care Med.

[33] Saldanha AJ (2004). Java Treeview–extensible visualization of microarray data.. Bioinformatics.

[34] Eisen MB, Spellman PT, Brown PO, Botstein D (1998). Cluster analysis and display of genome-wide expression patterns.. Proc Natl Acad Sci U S A.

[35] Fujita A, Sato JR, Demasi MA, Sogayar MC, Ferreira CE (2009). Comparing Pearson, Spearman and Hoeffding's D measure for gene expression association analysis.. J Bioinform Comput Biol.

[36] O'Neill LA (2008). Immunology. How frustration leads to inflammation.. Science.

[37] Suwa T, Hogg JC, Vincent R, Mukae H, Fujii T (2002). Ambient air particulates stimulate alveolar macrophages of smokers to promote differentiation of myeloid precursor cells.. Exp Lung Res.

[38] Newton R, Holden NS, Catley MC, Oyelusi W, Leigh R (2007). Repression of inflammatory gene expression in human pulmonary epithelial cells by small-molecule IkappaB kinase inhibitors.. J Pharmacol Exp Ther.

[39] Reibman J, Talbot AT, Hsu Y, Ou G, Jover J (2000). Regulation of expression of granulocyte-macrophage colony-stimulating factor in human bronchial epithelial cells: roles of protein kinase C and mitogen-activated protein kinases.. J Immunol.

[40] Stampfli MR, Wiley RE, Neigh GS, Gajewska BU, Lei XF (1998). GM-CSF transgene expression in the airway allows aerosolized ovalbumin to induce allergic sensitization in mice.. J Clin Invest.

[41] Gold JA, Hoshino Y, Tanaka N, Rom WN, Raju B (2004). Surfactant protein A modulates the inflammatory response in macrophages during tuberculosis.. Infect Immun.

