# Supplementary material for: Comparison of WTC Dust Size on Macrophage Inflammatory Cytokine Release In vivo and In vitro
Source: PLoS One. 2012 Jul 18;7(7):e40016. doi: 10.1371/journal.pone.0040016 (PMC3399845; doi:10.1371/journal.pone.0040016)
Supplement: Table S1 — Expression of Analytes by Alveolar Macrophages for All Stimuli A. Baseline Analyte Expression Above Limits of Detection (LOD) B. Baseline Analyte Expression Below LOD (DOCX) [file pone.0040016.s001.docx]

| **Supplemental Table 1A: Expression of Analytes by Alveolar Macrophages for All Stimuli***  **(Baseline Analyte Expression Above LOD)** | | | | | | | | |
| --- | --- | --- | --- | --- | --- | --- | --- | --- |
| **Analyte** | **MA** | **WTC PM_2.5_** | | | **WTC PM_10-53_** | | | **LPS** |
|  |  | **10 µg/mL** | **50 µg/mL** | **100 µg/mL** | **10 µg/mL** | **50 µg/mL** | **100 µg/mL** | **40 ng/ml** |
| **IFNα2** | 16.2(7-29) | 20.1(11-30) | 26.9(14-34) | 35.0(20-42) | 36.1(21-46) | 44.6(18-83) | 42.7(18-49) | 46.4(35-112) |
| **IFNγ** | 12.2(<3.2-16) | 11.5(7-22) | 23.0(9-31) | 26.2(11-33) | 31.8(15-43) | 33.6(19-57) | 36.0(17-62) | 53.5(37-86) |
| **IL-1α** | 4.3(<3.2-29) | 8.3(<3.2-31) | 11.0(<3.2-42) | 14.3(6-39) | 19.3(6-50) | 50.8(9-78) | 40.2(8-72) | 49.8(7-75) |
| **IL-1ra** | 177.5(43-751) | 206.6(36-410) | 318.4(54-693) | 404.3(65-1183) | 344.1(40-1037) | 352.2(48-1110) | 360.5(52-1235) | 412.7(55-1169) |
| **GM-CSF** | 16.4 (7-67) | 37.7(11-67) | 52.8(30-212) | 69.7(45-235) | 113.6(56-1274) | 305.9(125-3194) | 371.5(149-1082) | 267.5(163-1224) |
| **IP-10** | 53.8(20-1215) | 50.7(21-716) | 60.3(27-1246) | 28.2(14-311) | 371.1(41-1140) | 228.0(37-731) | 136.2(33-290) | 2587.1(39->10000) |
| **IL-6** | 32.0(8-62) | 83.1(14-123) | 115.5(58-211) | 239.2(131-489) | 708.8(191-1482) | 1309.1(385-1978) | 1409.6(404-2357) | 6148.2  (1978->10000) |
| **IL-10** | 11.1(<3.2-20) | 10.5(4-39) | 18.5(5-46) | 27.7(8-36) | 56.6(22-100) | 91.0(37-187) | 100.6(29-238) | 484.0(272-1186) |
| **TNF-α** | 31.6(13-142) | 57.4(18-98) | 139.1(97-234) | 340.8(233-689) | 945.8(349-1876) | 2178.6(1364-6640) | 1784.2(1209-9314) | 7852.7  (1803->10000) |
| **GRO** | 187.3(97-668) | 252.7(77-440) | 497.6(163-943) | 500.5(309-2297) | 1688.3(233-7429) | 1946.2(290-6226) | 600.2(75->10000) | 7852.7  (1803->10000) |
| **IL-7** | 20.7(<3.2-37) | 31.3(<3.2-46) | 23.5(<3.2-48) | 32.5(12-63) | 51.7(25-86) | 67.5(37-96) | 66.7(41-107) | 97.0(66-131) |
| **IL-8** | 3081.1  (1643-9115) | 5366.8  (2014-9379) | >10000.0  (2163->10000) | >10000.0  (2158->10000) | >10000.0  (1977->10000) | >10000.0  (2285->10000) | >10000.0  (2535->10000) | >10000.0  (2082->10000) |
| **IL-12 (p70)** | 3.4(<3.2-8) | 3.5(<3.2-8) | 4.6(<3.2-9) | 6.8(<3.2-9) | 9.1(4-11) | 9.2(6-11) | 9.0(5-10) | 11.1(7-20) |
| **Eotaxin** | 24.6(17-41) | 31.1(22-43) | 31.2(24-41) | 38.5(26-46) | 38.6(27-45) | 40.9(28-47) | 37.8(23-45) | 41.2(28-51) |
| **Flt-3 Ligand** | 10.4(5-14) | 11.1(6-19) | 12.8(9-30) | 17.0(9-38) | 17.2(8-42) | 16.1(9-46) | 12.9(7-35) | 21.4(6-46) |
| **Fractalkine** | 33.5(30-69) | 48.3(35-85) | 55.4(41-79) | 88.8(54-112) | 92.7(60-154) | 131.7(81-186) | 147.6(63-192) | 253.7(150-292) |
| **sIL-2Rα** | <3.2(<3.2-6) | <3.2(<3.2-7) | <3.2(<3.2-8) | 6.6(<3.2-10) | 8.9(<3.2-13) | 9.2(<3.2-12) | 8.7(<3.2-14) | 11.7(7-17) |
| **MCP-1** | 2230.9  (1398-3138) | 2212.7  (1430-2612) | 2492.9  (1973-3385) | 2646.8  (2049-4777) | 2824.6  (2121-5587) | 2591.8  (1807-5486) | 2726.2  (2093-4160) | 3256.1  (2129-7128) |
| **MCP-3** | 221.9(95-309) | 215.2(93-385) | 238.1(54-375) | 86.5(23-282) | 264.0(134-604) | 240.9(73-518) | 196.4(21-422) | 635.0(115-3224) |
| **MIP-1α** | 96.6(44-824) | 158.1(72-856) | 383.4(345-3542) | 2920.1(769->10000) | 6091.3(254->10000) | 3575.7(666->10000) | 3028.2(43->10000) | >10000  (206->10000) |
| **MIP-1β** | 39.1(14-131) | 57.8(19-187) | 306.6(112-575) | 647.1(334-1619) | 926.3(61-2471) | 2314.7(112-3426) | 273.9(15-3528) | 8662.0(  1199->10000) |
| **MDC** | 1803.0(483-3348) | 2040.0(414-4786) | 2700.0(1229-7482) | 3236.7(974-5234) | 5667.2(777-8713) | 5072.6(1244->10000) | 5008.6(62->10000) | 7088.5  (1193->10000) |
| **^*^**Analytes reported as Median (IQR), pg/mL Analytes with median baseline expression above limits of detection | | | | | | | | |

| **Supplemental Table 1B: Expression of Analytes by Alveolar Macrophages for All Stimuli***  **(Baseline Analyte Expression Below LOD)** | | | | | | | | |
| --- | --- | --- | --- | --- | --- | --- | --- | --- |
| **Analyte** | **MA** | **WTC PM_2.5_** | | | **WTC PM_10-53_** | | | **LPS** |
|  |  | **10 µg/mL** | **50 µg/mL** | **100 µg/mL** | **10 µg/mL** | **50 µg/mL** | **100 µg/mL** | **40 ng/ml** |
| **IL-2** | <3.2(<3.2-8) | <3.2(<3.2-10) | 5.9(<3.2-13) | 5.0(<3.2-10) | 10.7(4-13) | 9.9(4-14) | 8.3(<3.2-13) | 4.1(<3.2-12) |
| **IL-3** | <3.2(<3.2-8) | <3.2(<3.2-12) | <3.2(<3.2-12) | <3.2(<3.2-8) | <3.2(<3.2-12) | <3.2(<3.2-8) | <3.2(<3.2-8) | <3.2(<3.2-16) |
| **IL-4** | <3.2(<3.2-5) | <3.2(<3.2-7) | <3.2(<3.2-9) | <3.2(<3.2-9) | <3.2(<3.2-10) | <3.2(<3.2-10) | <3.2(<3.2-10) | 6.2(<3.2-12) |
| **IL-5** | <3.2(<3.2-3.2) | <3.2(<3.2-<3.2) | <3.2(<3.2-<3.2) | <3.2(<3.2-<3.2) | <3.2(<3.2-<3.2) | <3.2(<3.2-<3.2) | <3.2(<3.2-<3.2) | <3.2(<3.2-<3.2) |
| **IL-9** | <3.2(<3.2-3.2) | <3.2(<3.2-<3.2) | <3.2(<3.2-<3.2) | <3.2(<3.2-<3.2) | <3.2(<3.2-<3.2) | <3.2(<3.2-<3.2) | <3.2(<3.2-<3.2) | <3.2(<3.2-<3.2) |
| **IL-12 (p40)** | <3.2(<3.2-7) | <3.2(<3.2-10) | 7.1(<3.2-11) | 10.2(5-20) | 12.0(5-28) | 14.6(4-21) | 15.9(<3.2-33) | 104.8(51-157) |
| **IL-13** | <3.2(<3.2-4) | <3.2(<3.2-5) | <3.2(<3.2-5) | <3.2(<3.2-6) | 4.5(<3.2-7) | 6.4(<3.2-11) | 6.0(<3.2-12) | 3.9(<3.2-10) |
| **IL-15** | <3.2(<3.2-9) | <3.2(<3.2-9) | <3.2(<3.2-9) | <3.2(<3.2-9) | <3.2(<3.2-11) | <3.2(<3.2-11) | <3.2(<3.2-11) | <3.2(<3.2-14) |
| **IL-17** | <3.2(<3.2-11) | <3.2(<3.2-15) | <3.2(<3.2-12) | <3.2(<3.2-12) | 4.0(<3.2-18) | 4.7(<3.2-16) | 3.8(<3.2-14) | 5.0(<3.2-22) |
| **sIL-2Rα** | <3.2(<3.2-6) | <3.2(<3.2-7) | <3.2(<3.2-8) | 6.6(<3.2-10) | 8.9(<3.2-13) | 9.2(<3.2-12) | 8.7(<3.2-14) | 11.7(7-17) |
| **TGF-α** | <3.2(<3.2-3.2) | <3.2(<3.2-<3.2) | <3.2(<3.2-<3.2) | <3.2(<3.2-5) | 3.5(<3.2-18) | 8.1(<3.2-51) | 4.2(<3.2-43) | 6.6(<3.2-58) |
| **TNF-β** | <3.2(<3.2-12) | <3.2(<3.2-18) | <3.2(<3.2-18) | <3.2(<3.2-19) | <3.2(<3.2-22) | <3.2(<3.2-23) | <3.2(<3.2-18) | 4.6(<3.2-32) |
| **EGF** | <3.2(<3.2-3.2) | <3.2(<3.2-9) | <3.2(<3.2-6) | <3.2(<3.2-8) | <3.2(<3.2-13) | <3.2(<3.2-10) | <3.2(<3.2-11) | <3.2(<3.2-30) |
| **FGF-2** | <3.2(<3.2-10) | 6.6(<3.2-15) | 9.5(<3.2-14) | 12.2(<3.2-21) | 13.8(<3.2-25) | 12.7(<3.2-22) | 13.9(<3.2-28) | 32.9(11-221) |
| **sCD40L** | <3.2(<3.2-3.2) | <3.2(<3.2-<3.2) | <3.2(<3.2-5) | <3.2(<3.2-<3.2) | <3.2(<3.2-6) | <3.2(<3.2-8) | <3.2(<3.2-19) | 12.5(<3.2-43) |
| **IL-1β** | <3.2(<3.2-3.2) | <3.2(<3.2-8) | 3.4(<3.2-19) | 16.9(8-26) | 22.5 (8-59) | 36.2(24-104) | 50.0(24-146) | 56.0(39-132) |
| **G-CSF** | <3.2 (<3.2-18) | <3.2(<3.2-20) | 8.7(<3.2-23) | 27.6(<3.2-34) | 39.5 (<3.2-99) | 60.9(<3.2-339) | 103.7(12-390) | 957.4(162->10000) |
| ^*^Analytes reported as Median (IQR) in pg/ml. Analytes with median baseline expression below limits of detection | | | | | | | |  |
